# *Who* shows the Unlikelihood Effect – and *why*?

**DOI:** 10.3758/s13423-024-02453-z

**Published:** 2024-01-29

**Authors:** Moritz Ingendahl, Johanna Woitzel, Hans Alves

**Affiliations:** Department of Psychology, Ruhr University Bochum, Germany, Universitätsstraße 150, D-44801 Bochum, Germany

**Keywords:** Unlikelihood effect, Probability judgments, Risk, Uncertainty, Cognitive bias

## Abstract

Recent work shows that people judge an outcome as less likely when they learn the probabilities of all single pathways that lead to that outcome, a phenomenon termed the *Unlikelihood Effect*. The initial explanation for this effect is that the low pathway probabilities trigger thoughts that deem the outcome unlikely. We tested the alternative explanation that the effect results from people’s erroneous interpretation and processing of the probability information provided in the paradigm. By reanalyzing the original experiments, we discovered that the Unlikelihood Effect had been substantially driven by a small subset of people who give extremely low likelihood judgments. We conducted six preregistered experiments, showing that these people are unaware of the total outcome probability and do formally incorrect calculations with the given probabilities. Controlling for these factors statistically and experimentally reduced the proportion of people giving extremely low likelihood judgments, reducing and sometimes eliminating the Unlikelihood Effect. Our results confirm that the Unlikelihood Effect is overall a robust empirical phenomenon, but suggest that the effect results at least to some degree from a few people’s difficulties with encoding, understanding, and integrating probabilities. Our findings align with current research on other psychological effects, showing that empirical effects can be caused by participants engaging in qualitatively different mental processes.

**Supplementary Information:**

The online version contains supplementary material available at 10.3758/s13423-024-02453-z.

## Introduction

Risk assessment is a fundamental task in everyday life, from health to financial decisions. Yet, people often struggle with assessing risks adequately (Gigerenzer et al., 2007; Hoffrage et al., 2000). Recently, Karmarkar and Kupor (2023) discovered a new bias in people’s risk judgments – the *Unlikelihood Effect*. When people learn the multiple pathways and associated probabilities leading to a risk, they underestimate the risk’s likelihood. For example, imagine that there is a 58% chance of getting an infection from a flea bite. Receiving additional information that 10% of people get it from a siphonaptera flea, 8% from a psoroph flea, etc., reduces one’s subjective likelihood of the risk.

Karmarkar and Kupor (2023) demonstrated this Unlikelihood Effect in 13 experiments with different scenarios, probabilities, and measures. All experiments had a single-probability condition where participants were informed of the total outcome probability (TOP; e.g., 58%) and a multiple-probabilities condition where participants received (additional) information about each possible cause and its probability. In all experiments, participants gave lower subjective likelihood judgments if they learned the multiple probabilities. The explanation offered by Karmarkar and Kupor (2023) was that exposure to the low pathway probabilities triggers thoughts that the outcome is unlikely. In the same way that a message generates favorable/unfavorable thoughts and thus changes attitudes (Briñol & Petty, 2009), low probabilities should trigger “likely”/”unlikely” thoughts and change perceptions of the outcome’s likelihood.

In the present research, we further elaborate on the processes underlying the Unlikelihood Effect. We propose and test an alternative explanation: Some people deviate from probability calculus and therefore give formally incorrect judgments.

## Probability calculus and the Unlikelihood Effect

Much research has shown that people struggle with processing statistical information (Alves & Mata, 2019; Khemlani et al., 2015; Tversky & Kahneman, 1983), especially probabilities. Famous examples are the conjunction fallacy (Tversky & Kahneman, 1983) and probability matching (e.g., Gaissmaier & Schooler, 2008), among others. To illustrate how people might fail with probability calculus in the Unlikelihood Effect, consider the tasks from Experiment 1 by Karmarkar and Kupor (2023). In the single-probability condition, participants received the information: “Every single person has a 58% chance of getting a flea bite that causes a newly discovered bacterial infection. Specifically: 58% of people get this bacterial infection from getting bitten by a siphonaptera flea.” In the multiple-probabilities condition, the last sentence was replaced with seven sentences stating “8% of people get this bacterial infection from getting bitten by a culex flea; 10% of people get this bacterial infection from getting bitten by a aedes flea. […]”. All participants were asked: “In total, how likely are people to get this bacterial infection?” and presented with a response slider from “not likely at all” to “extremely likely.”

In both conditions, formal probability calculus (Kolmogoroff, 1933) prescribes a judgment of 58%, which is the probability of getting the infection. The flea type is actually irrelevant. If people follow formal probability calculus, they should rely exclusively on the 58% and translate it into a value on the response slider^1^ (cf. Windschitl, 2002). However, two asymmetries between the conditions make a judgment consistent with probability calculus less likely in the multiple-probabilities scenario: awareness of the TOP and required knowledge of probability calculus.

### Awareness of total outcome probability (TOP)

In the single probability condition, the TOP (58%) is the only information given. In the multiple-probabilities condition, much judgment-irrelevant information is presented, distracting from the TOP. Furthermore, a closer look at the study materials shows that the TOP was not mentioned in the multiple-probabilities conditions in five experiments. In two other experiments, it had only been disclosed on pages before the lower probabilities and the judgment task. Thus, people in the multiple-probabilities condition are less likely to be aware of the TOP.

### Understanding probability calculus

People in the multiple-probabilities condition could still compute TOP by aggregating the pathway probabilities. However, there is only one formally correct aggregation – computing the *sum* (58%) of all lower probabilities. Other types of aggregation, such as the mean (8.3%) or mode (7%/8%/9%), lead to a too low TOP. People especially tend to average probabilities (Budescu & Yu, 2007; Mislavsky & Gaertig, 2022), which might make some people believe that the TOP of getting the infection is only around 10%.

Even if people actually read the TOP, they must actively ignore the multiple probabilities. Ignoring information is cognitively challenging and violates basic conversational rules (Englich et al., 2006; Grice, 1975; Ross et al., 1975). People use numerical information once provided, even if it is unrelated to the formally correct answer (Lawson et al., 2022). Furthermore, the multiple-probability information might lead to a different interpretation of the scenario, such as “Every single person has a 58% chance of getting a flea bite that *could* cause a bacterial infection.” Accordingly, some people might also interpret the low-path probabilities as conditional probabilities (i.e., 8% of 58%) and integrate them with the actual TOP. Again, this would lead to participants believing the TOP is lower than 58%.

To conclude, there is an asymmetry between the two conditions in how easily people could arrive at a judgment in line with formal probability calculus, which may contribute to the Unlikelihood Effect. Our explanation allows the following predictions:

#### Quantitative versus qualitative differences

The Unlikelihood Effect scenarios have a formally correct answer. If all participants solved them correctly, the likelihood judgments should be around the TOP. Apart from some random noise introduced by the imprecise slider, the task is essentially an all-or-nothing task similar to the often-used bat-and-ball problem. Participants get it either right or wrong, engaging in qualitatively different cognitive actions. If the multiple-probabilities condition has a higher chance of errors, more participants will fall outside of the distribution around the correct value, and the distribution will become multimodal. Hence, we expect the Unlikelihood Effect to be driven by a few participants providing rather extreme values and not by a symmetrical shift in mean values.

#### Awareness of the TOP

We predict that some people are unaware of the TOP, even if explicitly stated, leading to formally incorrect and mostly lower likelihood judgments in the multiple-probabilities condition. Excluding participants unaware of the TOP should therefore reduce the Unlikelihood Effect.

#### Improving understanding

We predict that some people engage in formally incorrect interpretations and calculations with the probabilities. Thus, all interventions targeting participants’ understanding should reduce the Unlikelihood Effect. For example, because visualizations increase people’s understanding of probabilities (e.g., Brase, 2009; Spiegelhalter et al., 2011), presenting a Venn diagram should lead to a better understanding and reduce the Unlikelihood Effect. Alternatively, practicing necessary math operations beforehand can improve understanding of the actual scenario (Pan & Rickard, 2018).

## Overview of the present research

We expect that some people are unaware of the TOP and do formally incorrect operations with the pathway probabilities. To test this, we first reanalyzed all experiments by Karmakar and Kupor (2023) to search for qualitative differences in the Unlikelihood Effect. Next, we conducted six preregistered experiments. In Experiment 1, we asked participants to explain their judgment and coded the answers regarding awareness of the TOP and understanding. In Experiments 2a–c, we tested three ways to reduce the Unlikelihood Effect – a memory check for the TOP, visualization, and a preceding math task. Experiments 3 and 4 tested these interventions in different scenarios to ascertain generalizability.^2^

## Re-analysis of Karmarkar and Kupor (2023)

### Method

We downloaded all data from the paper’s openly accessible researchbox folder (https://researchbox.org/451). We applied the same exclusion criteria as in the original studies. We restricted our re-analysis to the single-probability and multiple-probabilities conditions, although some experiments implemented additional between-subjects conditions. In all studies, we first reproduced the original result reported in the paper (see Table S1 in the Online Supplementary Material (OSM) for an overview). Next, we inspected the distributions of the data visually.

**Fig. 1:**
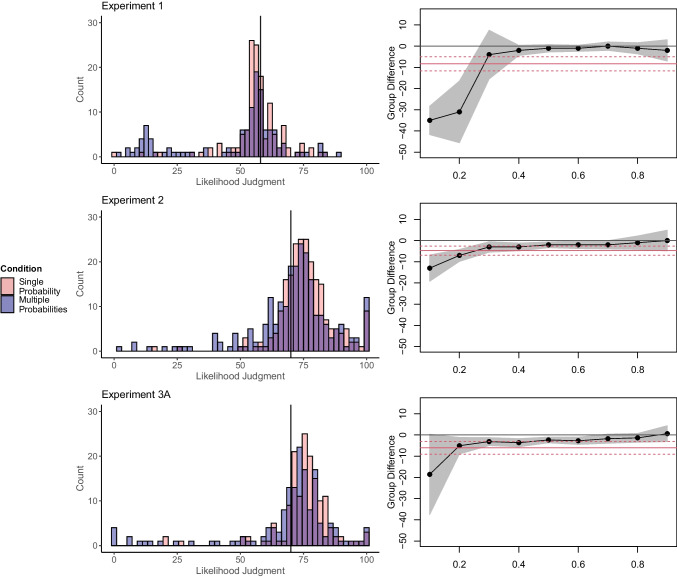

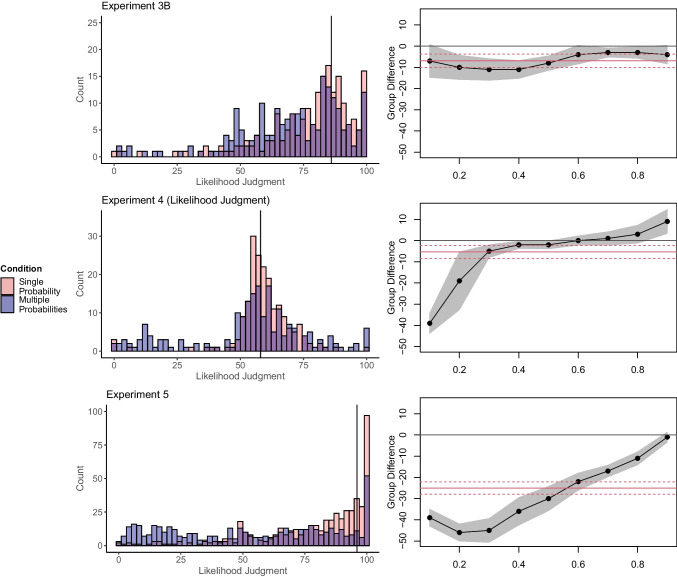
Likelihood judgments and quantile regressions from experiments of Karmarkar and Kupor (2023). Vertical lines in the histograms mark the total outcome probability (TOP) on the scale as a reference. In the quantile regression plots, red lines represent the mean difference with the 95% confidence interval (CI). Shaded areas represent 95% CIs of the quantile regression estimated via bootstrapping. We did not conduct quantile regressions on dependent variables that were not continuous, such as the proportion of generated thoughts in Experiment 4 or the decision of how much of a prevention treatment the participants would like to purchase from Experiment 6. However, we nevertheless inspected the distribution of these variables. Specifically, in Experiment 4, participants had to list their thoughts and categorize them regarding whether they focused on the outcome being likely, unlikely, or thoughts unrelated to the outcome’s likelihood. Seventy-seven percent of all participants listed zero unlikely thoughts. A small proportion (~5%) listed exclusively unlikely thoughts, with an unknown number of total thoughts listed. Excluding them eliminated the Unlikelihood Effect (see OSM Supplement C for detailed results and visualization of the distribution). In Experiment 6, participants had to decide how much of a prevention treatment they would like to purchase. Over 50% of the participants chose the maximum value on that scale. We present corresponding visualizations in the OSM. We also present visualizations for the supplementary experiments SA, SB1, SB2, SC, SD, and SE in OSM Supplement A

Then, we conducted quantile regression within each experiment with the *quantreg* package in R (Koenker, 2022). Quantile regression allows estimating the effect of the manipulation on different quantiles (instead of the mean) of the dependent variable. For example, one could compare the difference between the two conditions for the 10%, 20%, etc. quantile. This approach can reveal whether the effect of the manipulation is different for different quantiles and possibly driven by a few participants that deviate substantially from the TOP. If the Unlikelihood Effect is driven by a few participants in the multiple-probability condition, quantile regression will show a strong effect for lower quantiles, but no or a small effect for higher quantiles. Figure 1 illustrates effect estimates as a function of the quantile together with 95% rank confidence intervals. Red lines visualize the mean difference with the 95% confidence interval. Detailed statistics of these regressions are provided on the OSF. A detailed explanation of this quantile regression for Experiment 1 from Karmarkar and Kupor (2023) is provided in OSM Supplement B.

### Results

In Experiments 1, 2, 3A, 4, SA, SB1, SB2, and SC responses in the multiple-probabilities condition were multimodal (Fig. 1). Whereas most participants gave judgments near the corresponding TOP, a small proportion gave very low judgments. In all experiments except 3B and SE, the effects were much stronger for lower than for higher quantiles. For example, in Experiment 1, the effect was strong for the 10% and the 20% percentiles but vanished for higher quantiles.

### Discussion

Re-analyses of Karmarkar and Kupor (2023) suggest that the Unlikelihood Effect was substantially but not exclusively driven by few participants. Whereas most participants gave judgments near the corresponding TOP, a few participants in the multiple-probabilities condition gave very low judgments. Quantile regressions show that group differences primarily occur for the lowest 10–30% quantiles but decrease or vanish for higher quantiles.

Such a pattern fits our explanation, but it cannot test that these participants are indeed unaware of the TOP or deviate from probability calculus. Therefore, we tested this by replicating Experiment 1 from Karmarkar and Kupor (2023) and letting participants explain their judgments.

## Experiment 1

### Methods

All data, analysis code, and research materials are in an OSF directory (10.17605/OSF.IO/6PFVG). This experiment was preregistered prior to conducting the research at https://aspredicted.org/TD6_16S.^3^

#### Design and participants

Our Experiment 1 was similar to Experiment 1 in Karmarkar and Kupor (2023), but included only the critical multiple-probabilities and single-probability conditions. In the original study, the effect size was *d* = 0.51. Replicating such an effect with 90% power required a sample size of 172 participants. We collected data from *N* = 200 English native speakers from the USA and the UK on Prolific Academic (134 female, 64 male, two prefer not to say; *M*_*age*_ = 41.01 years).

#### Procedure and materials

The experiment employed the flea scenario described above, with the same instructions and materials as Experiment 1 of Karmarkar and Kupor (2023). Participants first learned that the different fleas were present in all parts of the world. This information is necessary to determine the pathway probabilities as irrelevant. Participants were asked a question demonstrating that they understood this information. Next, participants received the actual infection scenario in line with their assigned conditions. As in the original study, the slider coded values from 0 to 100, but no numeric information was displayed.

On the next page, participants were told that they had given a response of XX [the participant’s value] on the slider that ranged from 0 to 100. We asked participants why and how they came to this judgment, and stated that there were no correct or incorrect answers. Participants could type their answers into a text box. Detailed instructions are on the OSF.

As in the original study, participants then completed an attention check: “In the information you read, what kind of animal bite would cause a bacterial infection?” As in the original study, eight participants did not correctly answer which type of animal the scenario was about and were excluded from all analyses.

#### Coding

We present exemplary explanations by participants in Table S5 in the OSM for illustration and the full data in the OSF directory. We first assessed whether participants mentioned the TOP of 58% in their explanations by coding whether the number occurred in the text. One of the authors coded whether participants’ answers indicated that they did not understand the formally correct way to solve the task in line with probability calculus. A research assistant who was unaware of the experiment’s purpose and hypothesis served as the second coder. The exact coding instructions are provided on the OSF. Inter-rater reliability was high (kappa = .60). Overall, participants’ answers indicated many deviations from a formally correct solution. Some participants reported a mathematical calculation inconsistent with formal probability calculus, such as computing a mean (instead of the sum) of the low-path probabilities (see Table S5 in the OSM). Others applied real-world knowledge that had not been mentioned in the scenario description, such as that some people might not report the infection. Note that such reasoning is not wrong, but inconsistent with formal probability calculus, which is why we also coded these answers as incorrect. For 47/192 participants, at least one coder stated that a participant did not understand the formally correct way to solve the task. For 24/192, both raters agreed.

### Results

**Fig. 2:**
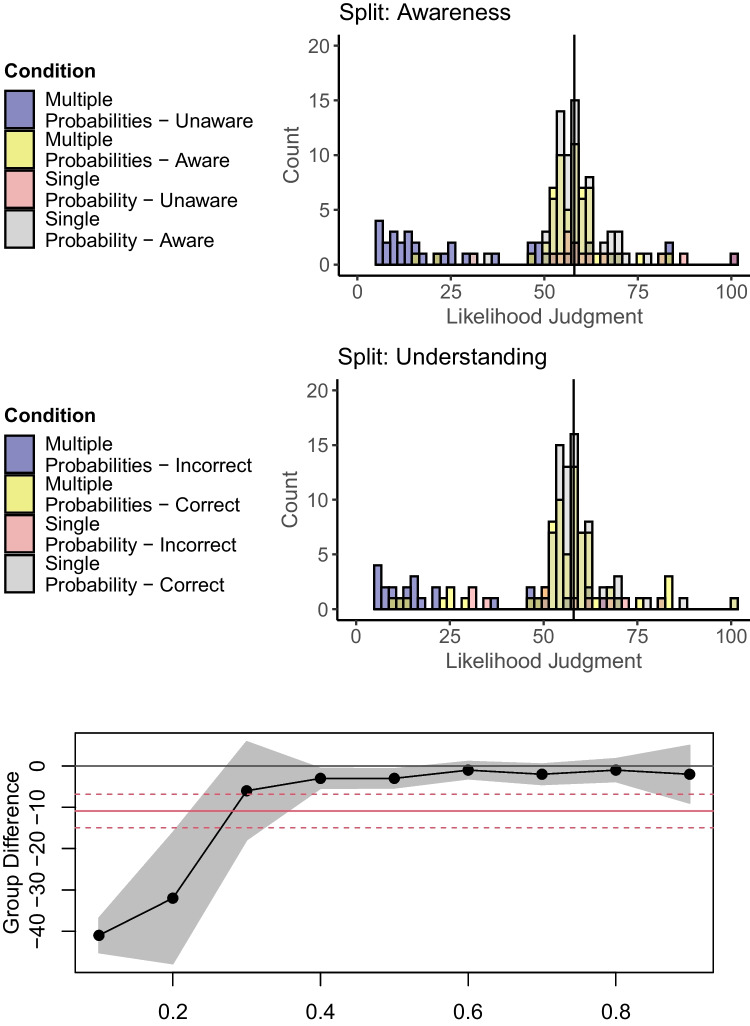
Likelihood judgments in Experiment 1 split by awareness of the total outcome probability (TOP) and correct understanding. Vertical lines mark the TOP on the scale as a reference. Note that the quantile regression was not preregistered in this experiment

Replicating the Unlikelihood Effect, likelihood judgments were overall lower in the multiple-probabilities condition. As in the original experiment, most participants in the multiple-probabilities condition gave judgments around 58%, but few participants chose a lower value (Fig. 2).

Participants mentioned the TOP in their explanations less often in the multiple-probabilities (61%) than in the single-probability condition (84%), χ^2^(1) = 11.71, *p* < .001. A Condition (Single-Probability vs. Multiple-Probabilities) × Mentioning (Yes vs. No) ANOVA showed a main effect of Condition, *F*(1, 188) = 32.93, *p* < .001, η^2^_p_ = .149, a main effect of Mentioning, *F*(1, 188) = 13.84, *p* < .001, η^2^_p_ = .069, and an interaction, *F*(1, 188) = 29.31, *p* < .001, η^2^_p_ = .135. Participants who did not mention the TOP showed a strong Unlikelihood Effect, *t*(188) = 6.41, *p* < .001, *d* = 1.17, CI_95%_ [0.53, 1.81], BF_10_ = 81.12. Participants who mentioned the TOP showed no effect, *t*(188) = 0.33, *p* = .741, *d* = 0.10, CI_95%_ [-0.24, 0.44], BF_10_ = 0.21.

Misunderstandings were also more frequent in the multiple-probabilities condition, χ^2^(1) = 5.01, *p* = .025. A Condition (Single-Probability vs. Multiple-Probabilities) × Understanding (Incorrect vs. Correct) ANOVA showed a main effect of Condition, *F*(1, 188) = 26.49, *p* < .001, η^2^_p_ = .123, a main effect of Understanding, *F*(1, 188) = 27.08, *p* < .001, η^2^_p_ = .126, and an interaction, *F*(1, 188) = 13.24, *p* < .001, η^2^_p_ = .066. Participants with incorrect understanding showed a strong Unlikelihood Effect, *t*(188) = 4.62, *p* < .001, *d* = 1.61, CI_95%_ [0.56, 2.64], BF_10_ = 14.26. For participants with correct understanding, the effect was smaller, *t*(188) = 2.42, *p* = .016, *d* = 0.40, CI_95%_ [0.09, 0.70], BF_10_ = 3.39.^4^

### Discussion

Experiment 1 confirmed that participants who misunderstood the scenario or did not mention the TOP showed a robust Unlikelihood Effect.^5^ Although these data support our explanation, Experiment 1 offers only correlative evidence. Participants might have justified their low judgments post hoc, leading to formally incorrect answers. Also, although we avoided the word “probability” when presenting participants their prior judgment, the numeric format might have made participants think more about the probabilities than for the initial judgment. In the following experiments, we therefore added minor modifications to the information presented to increase participants’ understanding.

## Experiments 2a–c

### Method

#### Design and participants

The following experiments were again direct replications of Experiment 1 by Karmarkar and Kupor (2023), only with the critical single-probability and the multiple-probabilities condition, but with minor modifications in Experiments 2b–c. Experiment 2a was a high-powered replication of Experiment 1 of Karmarkar and Kupor (2023) as a control baseline. In Experiment 2b, we added a pie chart to visualize the different probabilities. In Experiment 2c, we let participants first solve a math task similar to the reported scenario. In Experiments 2b–c, we also added a memory check for the TOP.

We decided to power each study generously with 400 participants to receive stable effect size estimates. The studies were conducted on the same platform and only separated by a few days of data collection. Although planned as individual experiments, we report them combined to facilitate comparisons of the different manipulations we administered. Each experiment was preregistered on aspredicted.com (2a: https://aspredicted.org/K41_SCZ, 2b: https://aspredicted.org/DWC_PHR, 2c: https://aspredicted.org/2X3_KYT).

We recruited *N* = 1,198 English native speakers (751 female, 443 male, three prefer not to say, three missing data; *M*_*age*_ = 40.43 years) from the UK and the USA via Prolific Academic. As preregistered (and done in the original experiment), we excluded all participants who failed to answer which animal the scenario dealt with (see the OSM Supplement D for the numbers).

#### Procedure and materials

##### Experiment 2a – baseline

Here, we used the same materials as Experiment 1 by Karmarkar and Kupor (2023). Thus, the procedure was identical to our Experiment 1, except that participants did not explain their judgment.

##### Experiment 2b – visualization aid and memory check

Experiment 2b was identical to Experiment 2a except for two changes: First, we added a pie chart with the different probabilities below the scenario description. Second, we added a memory check for the TOP after the likelihood judgment. Specifically, we presented participants with the following information: “On the previous page, we presented you with the following sentence: ‘Every single person has a **XX%** chance of getting a flea bite that causes a newly discovered bacterial infection. Specifically: …’ Which percentage was shown instead of the XX?” Participants could enter a whole number between 0 and 100 in an open response format.

##### Experiment 2c – math problem and memory check

Experiment 2c was identical to Experiment 2a except for two changes: First, we let participants solve a math problem before the actual task. Participants read: “There is a city called Springfield in the USA. 74% of the people living in Springfield own exactly one car, the rest of the people in Springfield do not own a car. Specifically: 8% of the people living in Springfield own a Ford. 11% of the people living in Springfield own a Honda. […] 3% of the people living in Springfield own a Tesla.” Participants were asked: “In total, how likely are the people in Springfield to own a car?” They had to type in a number from 0 to 100. Afterward, the task was identical to Experiment 2a. As in Experiment 2b, we also added the memory check for the TOP.

Note that in the supplementary experiments SB1 and SB2 of the original paper, the scenario also referred to the additive nature of the probabilities by stating: “Adding up the total probabilities [of colored marbles], 64% of people…”. The responses nevertheless showed a bimodal pattern, suggesting that participants either did not read this information presented at the bottom of the text or interpreted the “adding up” as a synonym for “in total” or “overall.” We therefore let participants explicitly sum up pathway probabilities so that they had to become aware of their additive nature.

### Results

**Fig. 3:**
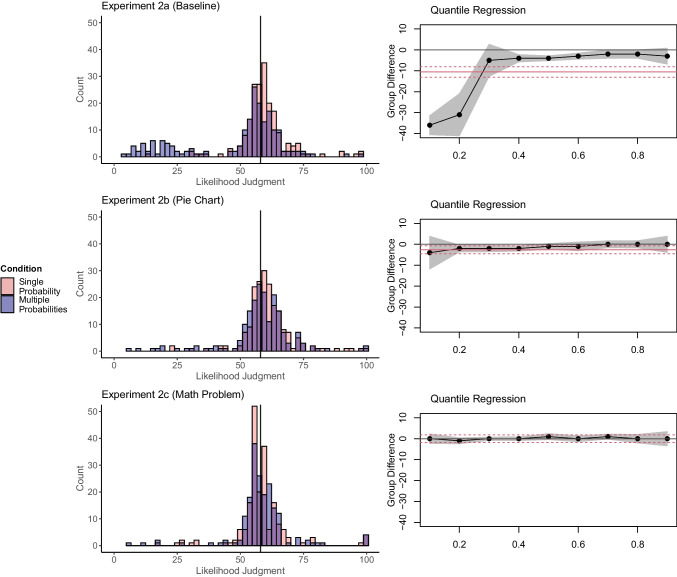
Distribution of likelihood judgments and quantile regressions in Experiments 2a–c. The vertical line represents the total outcome probability (TOP) on the subjective likelihood slider. Note that the quantile regressions were not preregistered in these experiments

Experiment 2a (Baseline) replicated the Unlikelihood Effect with a similar size to that in Experiment 1, *t*(390) = 6.97, *p* < .001, *d* = 0.70, CI_95%_ [0.50, 0.91], BF_10_ > 1000. Again, the multiple-probabilities condition had a bimodal distribution (see Fig. 3). In Experiment 2b (Pie Chart), the Unlikelihood Effect was weak, *t*(382) = 2.27, *p* = .024, *d* = 0.23, CI_95%_ [0.03, 0.43], BF_10_ = 1.34; excluding participants (Single: 26, Multiple: 58) who did not report the correct TOP eliminated the effect, *t*(298) = 0.66, *p* = .508, *d* = 0.08, CI_95%_ [-0.15, 0.31], BF_10_ = 0.16. In Experiment 2c (Math Problem), the effect was gone entirely, *t*(381) = 0.01, *p* = .992, *d* = 0.00, 95% CI [-0.20, 0.20], BF_10_ = 0.11. Excluding participants with an incorrect memory check (Single: 13, Multiple: 35) or an incorrect math problem answer did not change this (see the OSM Supplement D).

### Discussion

Experiments 2a–c again suggest that the Unlikelihood Effect is largely driven by a few participants who are unaware of the TOP or deviate from probability calculus. The effect was smaller or absent when presenting a pie chart or after letting participants do a math task requiring the necessary mental operations.

However, we used a scenario where the original experiment had shown a bimodal pattern in the multiple-probabilities condition. This was not the case in other scenarios, suggesting that other processes could exist. Also, in some experiments, the TOP was not disclosed in the multiple-probabilities condition. It would be helpful to see whether participants were nevertheless accurate in assessing the TOP. Lastly, we had tested the interventions with individual high-powered studies but not in a fully randomized experiment. Despite the parallels in the materials, data collection, etc., there might be unknown confounds.

We therefore conducted two further experiments with the interventions using other scenarios by Karmarkar and Kupor (2023), where the bimodal pattern did not emerge, and the TOP had not been disclosed.

## Experiment 3

### Methods

#### Design

Experiment 3 was a replication of Experiment 5 by Karmarkar and Kupor (2023), in which we also manipulated between participants whether a pie chart was shown or not, leading to a Condition (Single-Probability vs. Multiple-Probabilities) × Format (Pie Chart vs. Control) design. The original experiment had shown the strongest Unlikelihood Effect with *d* = 0.90. Replicating this effect with 90% power within each format condition required 54 participants (Faul et al., 2007). Because we expected that the pie chart would eliminate the effect, we expected a medium-sized interaction of *f* = .2 (Giner-Sorolla, 2018), which required *N* = 265 participants. Conservatively, we collected data from *N* = 400 English native speakers who were UK or US citizens from Prolific Academic (251 female, 148 male, one prefer not to say; *M*_*age*_ = 39.42 years). The experiment was preregistered on aspredicted.org (https://aspredicted.org/87Q_24B).

#### Materials and procedure

After giving informed consent, participants first had to pass a simple attention check where they had to type in the third word of the sentence, “a rolling stone gathers no moss.” Afterward, they read: “The vast majority of Americans do not consume enough Vitamin B12. Insufficient consumption of Vitamin B12 can harm the immune system. Insufficient Vitamin B12 will not impact with more than one protein in a single person’s body. Here’s how insufficient Vitamin B12 can harm the immune system:” In the single-probability condition, participants received the additional information: “Insufficient Vitamin B12 consumption harms the immune system in 96% of people by impacting the cytochrome protein.” In the multiple-probabilities condition, participants received 21 pieces of information in the style of “Insufficient Vitamin B12 consumption harms the immune system in XX% of people by impacting the YYYYY protein.” The probabilities at XX ranged from 2% to 8%. As in the original experiment, participants were not informed about the TOP in the multiple-probabilities condition.

Participants provided likelihood judgments on the same slider as in the other studies, and were asked, “In total, how likely is insufficient Vitamin B12 consumption to harm the immune system?” We did not assess the two items about behavioral intentions from the original experiment here. However, we assessed a memory check for the TOP on the next page. In the single-probability conditions, the wording was similar to the previous studies. In the multiple-probabilities conditions, the TOP had never explicitly been mentioned. Therefore, we asked: “On the previous page, we presented you with multiple sentences such as: ‘Insufficient Vitamin B12 consumption harms the immune system in XX% of people by impacting the YYYYY protein.’ What was the sum of all the probabilities mentioned in these sentences?” Because we expected nearly no participants to give the correct answer here, we preregistered that values +/-4 would still count as correct answers.

### Results

**Fig. 4:**
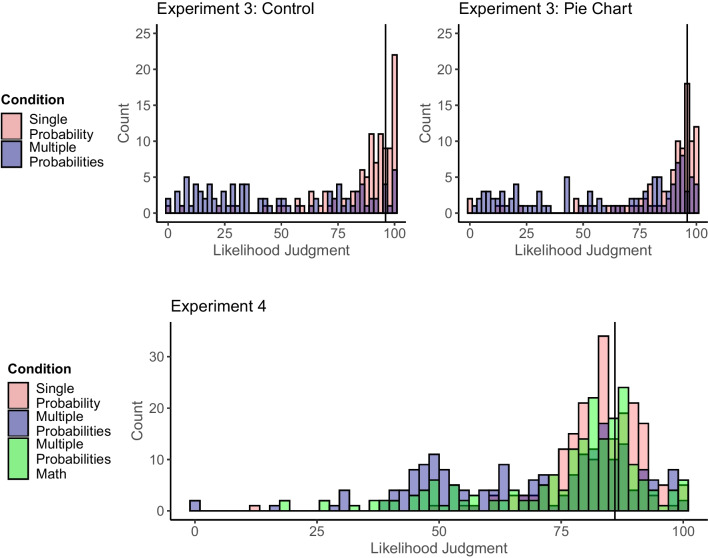
Likelihood judgments in Experiments 3 and 4. *Note.* The vertical line represents the total outcome probability (TOP) on the subjective likelihood slider. We present visualizations of the quantile regressions and the distributions after excluding participants in OSM Supplement D

We analyzed the judgments (Fig. 4) with a Condition (Single-Probability vs. Multiple-Probabilities) × Format (Pie Chart vs. Control) ANOVA. Next to a main effect of Condition, *F*(1, 398) = 125.90, *p* < .001, η^2^_p_ = .240, there was a significant interaction, *F*(1, 398) = 5.01, *p* = .026, η^2^_p_ = .012. For the control condition, there was a strong Unlikelihood Effect, *t*(398) = 9.56, *p* < .001, *d* = 1.39, CI_95%_ [1.08, 1.70], BF_10_ > 1000. For the pie chart condition, this effect was smaller but significant, *t*(398) = 6.32, *p* < .001, *d* = 0.87, CI_95%_ [0.57, 1.16], BF_10_ > 1000. Thirty-one (single-probability) and 81 participants (multiple-probabilities condition) were unaware of the TOP when using the preregistered^6^ +/-4 threshold. Following the preregistration, we repeated the analysis without these participants, reported in detail in OSM Supplement D. In essence, the Condition main effect was weaker and the interaction was no longer significant.

### Discussion

Experiment 3 replicated the effectiveness of the visualization intervention in a different scenario. However, the intervention effect was much weaker here than in Experiment 2, and a strong Unlikelihood Effect remained. The memory/awareness check indicated that most participants in the multiple-probabilities condition were unaware of the TOP. Excluding the participants unaware of the TOP reduced the effect; however, the degree of reduction depended on what still counted as a correct response.

## Experiment 4

### Methods

#### Design

Experiment 4 was a replication of Experiment 3B by Karmarkar and Kupor (2023) with an additional condition where participants first had to solve a math problem, leading to a unifactorial design with the conditions single probability, multiple probabilities, and multiple probabilities plus math. The original experiment had yielded an Unlikelihood Effect of *d* = 0.35. Replicating this effect with 90% power required 346 participants (Faul et al., 2007). Because we had an additional condition, we collected data from *N* = 604 English native speakers who were UK or US citizens from Prolific Academic (346 female, 252 male, two prefer not to say, two missing values; *M*_*age*_ = 41.02 years). The experiment was preregistered on aspredicted.org (https://aspredicted.org/DW8_YS5).

#### Materials and procedure

After giving informed consent, participants first had to pass a simple attention check where they had to type in the third word of the sentence “a rolling stone gathers no moss.” Four participants were excluded from all analyses due to incorrect answers here. Afterward, some participants had to solve a math task similar to the one in Experiment 2c but only with two probabilities. Next, all participants read that people have an 86% chance of experiencing a new pollen-induced allergic inflammation. Participants in the single-probability condition were then told that people had an 86% chance of experiencing this inflammation from breathing in the aika pollen. Participants in the other two conditions read that people had a 46% chance of experiencing this inflammation from breathing in the aika pollen and an additional 40% chance of experiencing this inflammation from breathing in the pola pollen.

Participants provided their likelihood judgments on the same slider as in the other studies, and were asked, “in total, how likely are people to experience this inflammation?” On the next page, we assessed the memory check for the TOP as in the previous studies. We preregistered that values +/-4 would still count as correct answers here.

### Results

We analyzed the judgments with an ANOVA, showing a significant effect, *F*(2, 598) = 34.37, *p* < .001, η^2^_p_ = .103. Likelihood judgments were lowest in the multiple-probabilities condition, higher in the math condition, and highest in the single-probability condition. Planned pairwise comparisons between all conditions were significant, Single vs. Multiple: *t*(598) = 8.29, *p* < .001, *d* = 0.85, CI_95%_ [0.64, 1.05], BF_10_ > 1000, Single vs. Math: *t*(598) = 3.94, *p* < .001, *d* = 0.37, CI_95%_ [0.18, 0.57], BF_10_ = 82.36, Math vs. Multiple: *t*(598) = 4.30, *p* < .001, *d* = 0.46, CI_95%_ [0.26, 0.66], BF_10_ > 1000. We also repeated the analysis without participants who were unaware of the TOP or did not solve the problem correctly. Again, we provide these analyses in OSM Supplement D. All mean differences were reduced but still significant.

### Discussion

Experiment 4 replicated the effectiveness of the math problem intervention in a different scenario. Different from Experiment 2c, the intervention only reduced the Unlikelihood Effect. Excluding participants unaware of the TOP reduced but did not eliminate differences between the conditions.

## General discussion

Learning the multiple pathways and probabilities leading to an outcome decreases the subjective likelihood of the outcome (Karmarkar & Kupor, 2023). We discovered that this *Unlikelihood Effect* was at least partially driven by a small proportion of participants giving very low judgments. In six experiments, we showed that some participants are unaware of the total outcome probability (TOP) and deviate from formal probability calculus. Helping participants understand the presented information reduces the Unlikelihood Effect.

Our research offers new theoretical insight into the cognitive mechanisms underlying the effect. Furthermore, our research suggests that there will be a substantial effect even if only a few people misunderstand the provided probability information. Risk communicators should therefore present multiple pathway probabilities in an easily accessible way (or not at all). Our research complements previous research, showing that people often struggle with interpreting probability information (Hertwig & Gigerenzer, 1999; Tversky & Kahneman, 1983). Further, deviations from formal standards might result because participants’ understanding of the given task differs from the experimenter's intended meaning (Dulany & Hilton, 1991; Schwarz et al., 1991). Like prior findings, our studies show that aiding comprehension through easy interventions like visualization (e.g., Brase, 2009; Spiegelhalter et al., 2011) or rehearsal of mathematical calculations (Pan & Rickard, 2018) can reduce biases.

Our interventions effectively reduced but did not always eliminate the Unlikelihood Effect, suggesting that the effect is also driven by other processes, such as “unlikely thoughts” triggered by the low probabilities, as suggested by Karmarkar and Kupor (2023). However, reanalyzing the primary experiment testing this explanation shows that only a minority generates these “unlikely thoughts” (see OSM Supplement C). Alternatively, the low pathway probabilities may lead participants to interpret the scale differently (e.g., Schwarz, 1999) and change what is considered extremely likely or unlikely. In addition, although support theory would generally predict higher likelihood judgments when participants are exposed to multiple pathways of an outcome (Tversky & Koehler, 1994), it predicts the opposite under specific conditions (Rottenstreich & Tversky, 1997) – for example, if participants “repack” highly similar pathways into an overall event. These different explanations deserve to be investigated in future research.

More generally, our research shows that the Unlikelihood Effect should not be understood as an *average treatment effect*. This adds insights to a current debate in cognitive psychology: to what extent qualitative differences emerge in established phenomena (Rouder & Haaf, 2021). For example, Schnuerch and colleagues (Schnuerch et al., 2021) demonstrated qualitative differences in the truth effect (for a meta-analysis, see Dechêne et al., 2010). Whereas most people are more likely to believe a statement encountered more often, some people systematically show the opposite effect (Schnuerch et al., 2021). Our research shows a similar pattern. Whereas most people’s judgments are close to the TOP, a few people’s judgments strongly diverge, indicating qualitatively different mental processes.

### Limitations and open questions

In the present work, we focused on situational factors moderating the Unlikelihood Effect, allowing internally valid tests and recommendations for practical applications like health communication. Yet, the substantial qualitative differences we find raise the question of whether individual-level predictors^7^ like numeracy (Peters et al., 2006) or cognitive reflection (Frederick, 2005) explain which individuals show the effect.

Additionally, our research shows that the Unlikelihood Effect can emerge due to deviations from formal standards. We identified some deviations, but there are a myriad ways in which one can err. Determining if the persistent Unlikelihood Effect in our studies originates from specific errors, cognitive biases, or “unlikely thoughts” from low probabilities is a topic for future studies.

## Conclusion

Our research provides a novel perspective on why people deem an event less likely when being informed about its pathways and associated probabilities. More generally, our research emphasizes that a robust effect can sometimes result from a few people engaging in qualitatively different mental processes.

### Supplementary Information

Below is the link to the electronic supplementary material.

Online Supplementary Material (PDF 1.18 MB)

## Data Availability

The data, analysis code, and materials for all experiments are available at 10.17605/OSF.IO/6PFVG . All experiments were preregistered, links to the preregistrations are provided in the respective study sections.

## Footnotes

1 In this research, we take formal probability calculus as a prescriptive norm from which some people deviate. Whether this prescriptive norm is reasonable or too strict for subjective likelihood judgments has been a debate in previous research (Gigerenzer, 1996; Kahneman & Tversky, 1996), which we do not want to reiterate here (for a review, see Vranas, 2000). For us, it only matters that there is a formally correct answer prescribed by probability calculus, people deviate from the prescriptive norm, and, furthermore, the extent to which people deviate from this prescriptive norm depends on several factors investigated in this research.

2 We conducted three supplementary experiments presented in OSM Supplement E. In Experiment SA, we used the scenario from Experiment 1 with a slightly different question and the memory check for the TOP in 2b/2c. The effect was as strong as in the baseline experiment 2a, but significantly weaker after excluding participants without memory of the TOP. In Experiment SB, we presented the scenario from Experiment 1 in a natural frequency format. This reduced but did not eliminate the effect compared to the baseline condition. In Experiment SC, we specifically tested the influence of averaging by asking participants two separate questions, “in total, how likely are people to get the infection *from a specific flea*?” and “in total, how likely are people to get the infection *from any type of flea*?” There was a strong effect on the first, but no effect on the second judgment.

3 In most experiments, we preregistered that we would also search visually for subclusters in the data and repeat the *t*-tests without subclusters. As this approach is not very objective, we switched to the quantile regressions, which do not require any exclusion of participants. We provide the results from our visual search for subclusters in OSM Supplement C.

4 We also conducted an ANOVA coding only those responses as incorrect where both raters agreed (see the R Markdown on the OSF for detailed results). This analysis showed the same effects except that the Unlikelihood Effect was not significant anymore for participants with correct understanding. In a final exploratory ANOVA, we added both understanding and TOP mentioning as moderators. Both two-way interactions (Understanding × Condition and Mentioning × Condition) were still significant, suggesting that they had independent effects.

5 As a side note, participants’ responses also revealed that many participants intended to give a judgment of 58%. Many participants complained that they missed the 58% simply because the slider did not show any numerical values.

6 Only eight participants in the multiple-probabilities conditions reported the correct number 96 here. Furthermore, 76 of 107 “correct” participants had responded with 100, which might also be the result of mere guessing. Therefore, we conducted an exploratory ANOVA without participants responding with 100, provided in the OSM. The Condition main effect was now gone, together with the interaction and the other main effect were also not significant. Note, however, that there were only 31 participants left in the multiple-probabilities conditions for this analysis.

7 Following the suggestion of an anonymous reviewer and previous research on gender differences in risk perception (Byrnes et al., 1999), we examined whether gender moderated the Unlikelihood Effect in Experiments 1 and 2a where we could match the sociodemographic data with the experimental data. We did not find a significant moderation by gender, *p*s > .208, and the Unlikelihood Effect was significant for female and non-female participants.
