# Supplementary material for: Who shows the Unlikelihood Effect – and why?
Source: Psychon Bull Rev. 2024 Jan 29;31(4):1768–81. doi: 10.3758/s13423-024-02453-z (PMC11358238; doi:10.3758/s13423-024-02453-z)
Supplement: Supplementary file 1 — (PDF 1.18 MB) [file 13423_2024_2453_MOESM1_ESM.pdf]

Supplement A: Full Reanalysis of Karmarkar & Kupor (2023)

Table S1

Re-Analysis of Karmarkar and Kupor (2023)

Exp. Nr.	Scenario	Outcome Probability	Low-Path Probabilities	Dependent Variable	Original Result	Total Outcome Probability Stated?
1	Flea	58%	Seven Probabilities (7-10%)	Slider (0-100)	$t(259) = 4.11$, $p < .001$, $d = 0.51$, 95% CI [0.26, 0.76], $BF_{10} = 336.84$	Yes
2	Cancer	70%	Six Probabilities (67%, 1%, 4 x 0.5%)	Slider (0-100)	$t(452) = 3.62$, $p < .001$, $d = 0.34$, 95% CI [0.15, 0.52], $BF_{10} = 55.32$	On the previous page
3A	Medication	70%/70.001%	70% + 0.001%	Slider (0-100)	$t(306) = 3.28$, $p = .001$, $d = 0.37$, 95% CI [0.15, 0.60], $BF_{10} = 20.21$	Yes
3B	Allergy	86%	40% + 46%	Slider (0-100)	$t(398) = 3.55$, $p < .001$, $d = 0.35$, 95% CI [0.16, 0.55], $BF_{10} = 44.33$	On the previous page
4	Medication	58%	Nine Probabilities (3-10%)	Slider (0-100)	$t(390) = 2.89$, $p = .004$, $d = 0.29$, 95% CI [0.09, 0.49], $BF_{10} = 6.00$	Yes
4	Medication	58%	Nine Probabilities (3-10%)	Proportion of Unlikely Thoughts	$t(383) = -2.11$, $p = .035$, $d = -0.22$, 95% CI [-0.42, -0.01], $BF_{10} = 0.96$	Yes
5	Vitamin	96%	21 Probabilities (2-8%)	Slider (0-100)	$t(998) = 14.24$, $p < .001$, $d = 0.90$, 95% CI [0.77, 1.03], $BF_{10} > 1000$	No
6	Flea	74%	Nine Probabilities (3-15%)	Choice 1- 11	$t(511) = 2.26$, $p = .024$, $d = 0.20$, 95% CI [0.03, 0.37], $BF_{10} = 1.17$	No
SA	Balls in Urn	60%	Eleven Probabilities (3-8%)	Slider (0-100)	$t(301) = 4.96$, $p < .001$, $d = 0.57$, 95% CI [0.34, 0.80], $BF_{10} > 1000$	No
SB1	Lottery Game	64%	Seven Probabilities (7-13%)	Slider (0-100)	$t(203) = 3.25$, $p = .001$, $d = 0.45$, 95% CI [0.18, 0.73], $BF_{10} = 19.48$	Yes
SB2	Lottery Game	64%	Seven Probabilities (7-13%)	Slider (0-100)	$t(301) = 2.15$, $p = .033$, $d = 0.25$, 95% CI [0.02, 0.47], $BF_{10} = 1.13$	Yes
SC	Medication	60%/61%	60% + 1%	Slider (0-100)	$t(420) = 3.32$, $p < .001$, $d = 0.32$, 95% CI [0.13, 0.51], $BF_{10} = 20.89$	Yes

Exp. Nr.	Scenario	Outcome Probability	Low-Path Probabilities	Dependent Variable	Original Result	Total Outcome Probability Stated?
SD	Allergy	40%	Five Probabilities (5-11%)	Slider (0-100)	$t(258) = 2.47$, $p = .014$, $d = 0.31$, 95% CI [0.06, 0.55], $BF_{10} = 2.39$	No
SE	Genes	27-28%	25%, 2-3%	Slider (0-100)	$t(392) = 2.37$, $p = .018$, $d = 0.24$, 95% CI [0.04, 0.44], $BF_{10} = 1.64$	No

Figure S1

Likelihood Judgments and Quantile Regressions from ALL Experiments of Karmarkar and Kupor (2023)

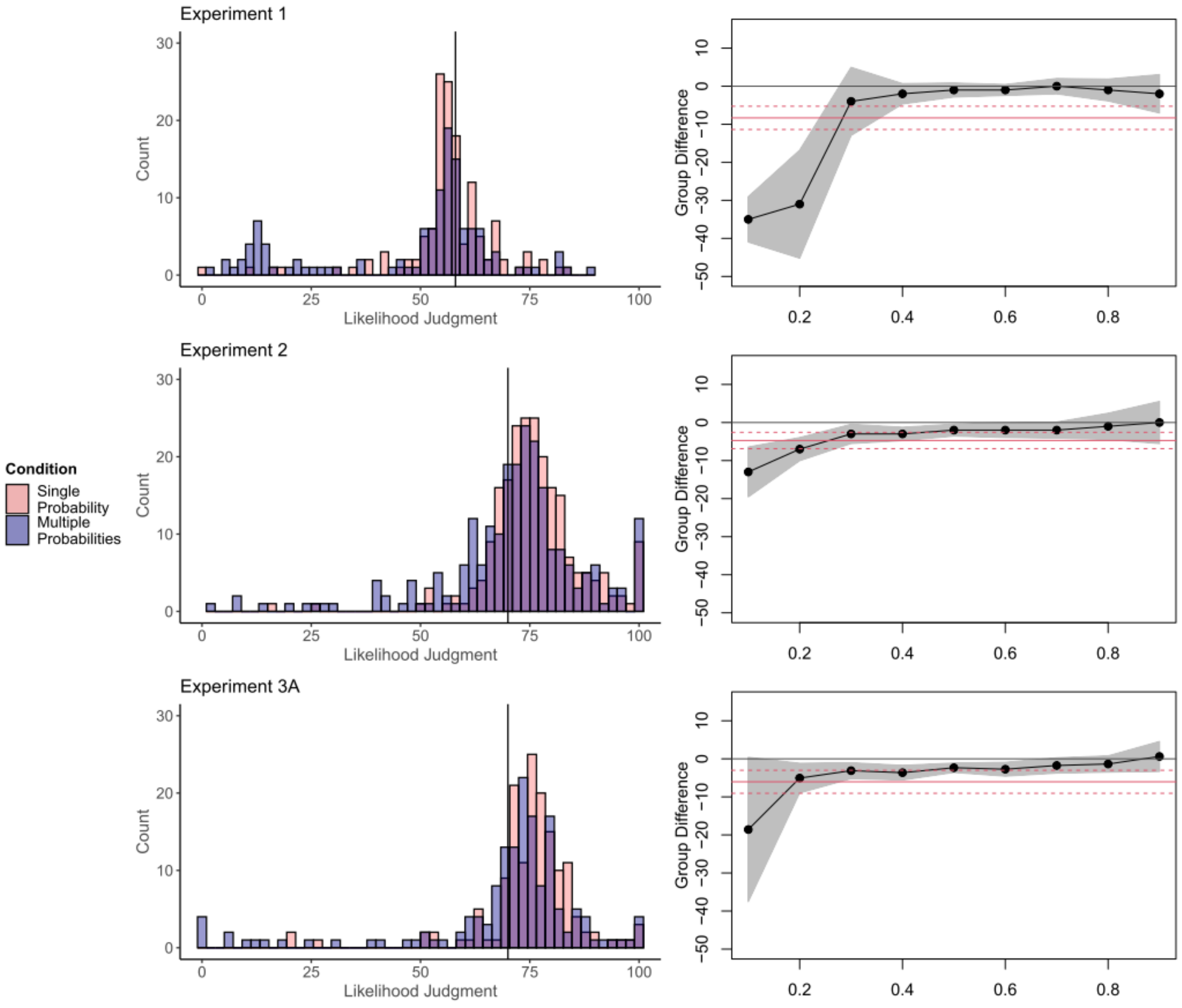

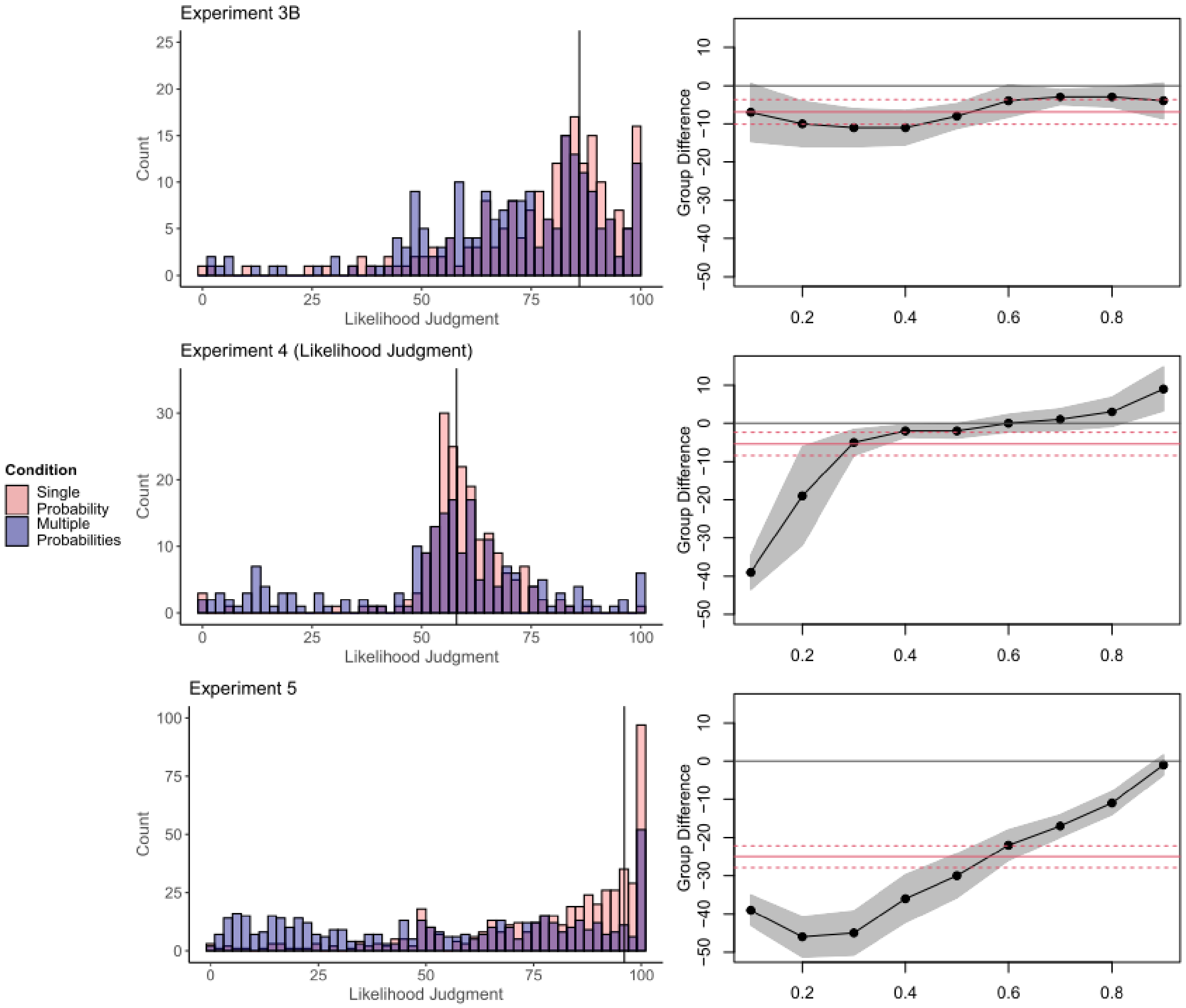

Experiment 3B
Count
Likelihood Judgment
Group Difference
Experiment 4 (Likelihood Judgment)
Condition
Single Probability
Multiple Probabilities
Experiment 5

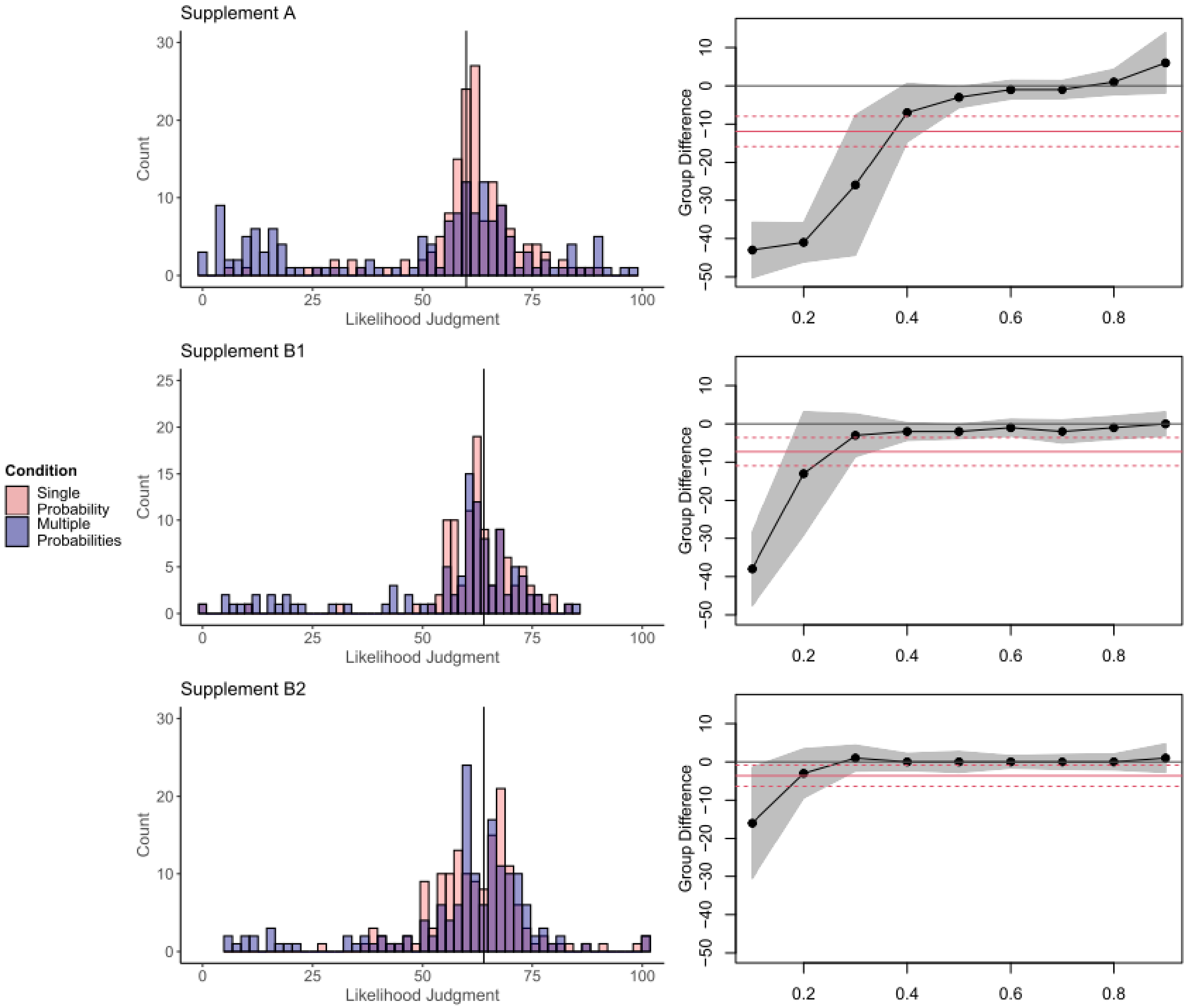
Supplement A
Count
Likelihood Judgment
Group Difference
Supplement B1
Count
Likelihood Judgment
Group Difference
Supplement B2
Count
Likelihood Judgment
Group Difference
Condition
Single Probability
Multiple Probabilities

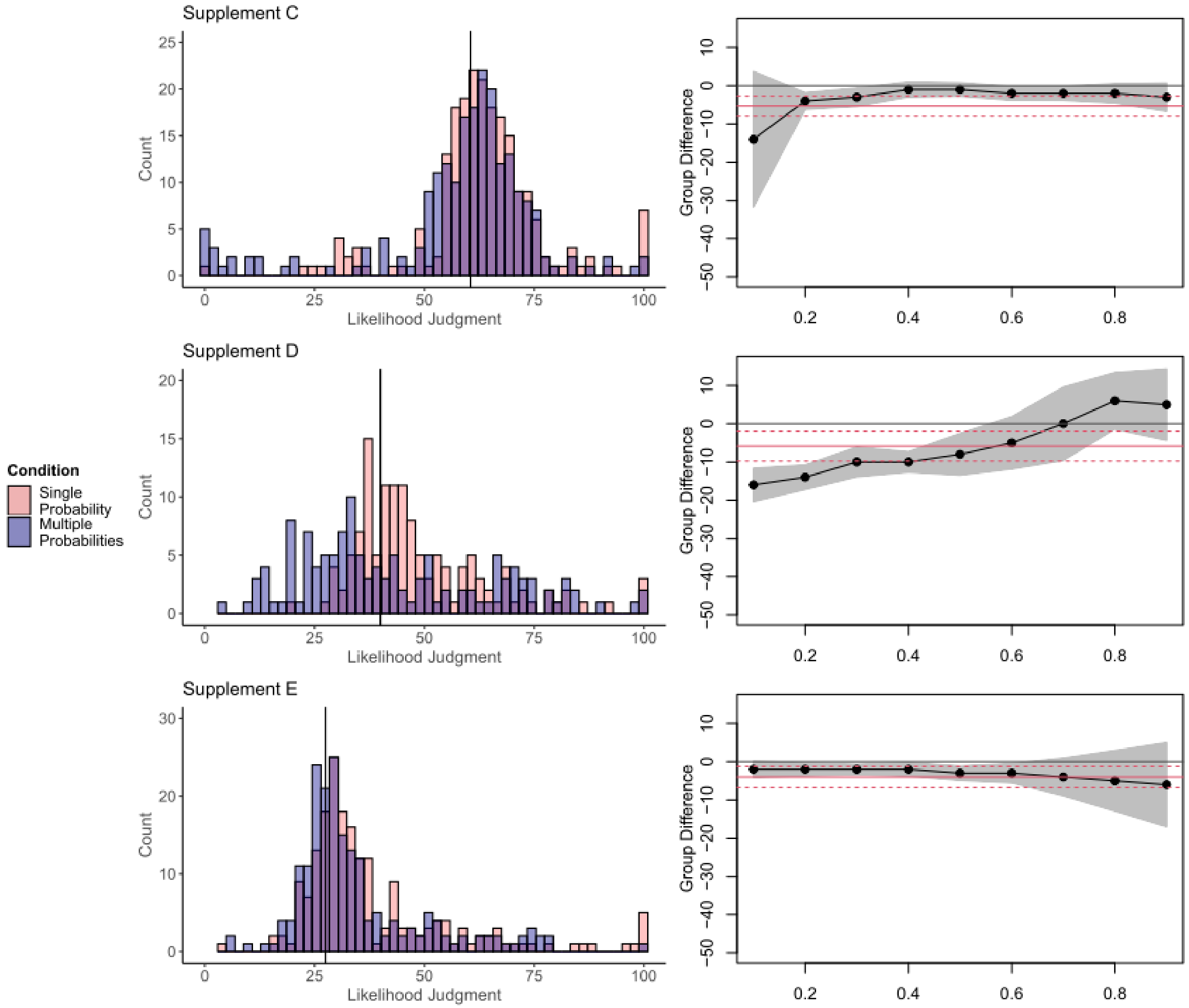
Supplement C
Supplement D
Supplement E
Count
Likelihood Judgment
Group Difference
Condition
Single Probability
Multiple Probabilities

Note. Vertical lines in the histograms mark the total outcome probability on the scale as a reference. In the quantile regression plots, red lines represent the mean difference with the 95% confidence interval. Shaded areas represent 95% confidence intervals of the quantile regression estimated via bootstrapping.

Supplement B: Explanation of Quantile Regression in Experiment 1 of Karmarkar & Kupor (2023)

We conducted quantile regressions within each experiment with the *quantreg* package in R (Koenker, 2022). Quantile regression allows estimating the effect of the manipulation on different quantiles (instead of the mean) of the dependent variable. In our case, we compared the difference between the two conditions for the 10%, 20%, etc. quantile. This approach can reveal whether the effect of the manipulation is different for different quantiles, thereby allowing a test of whether the effect on the mean difference is primarily driven by a few participants that deviate substantially from the total outcome probability. Suppose the Unlikelihood Effect is driven by a few participants in the multiple-probability condition giving extremely low judgments. In that case, quantile regression will show a strong effect for lower quantiles but no or a small effect for higher quantiles.

We present the quantile regression results of Experiment 1 of Karmarkar and Kupor (2023) in Table 1. For the 10% quantile, there was a strong difference in the direction of the Unlikelihood Effect between the two conditions, $\text{Quantile}_{10\_Single} = 47.00$, $\text{Quantile}_{10\_Multiple} = 12.00$, $t = -8.172$, $p < .001$. This difference was still significant for the 20% quantile, but not for any other quantiles of the dependent variable (see Table 1). Thus, the two conditions differed significantly only in the lower but not in the higher quantiles, suggesting that the effects of the multiple-probabilities manipulation made a few participants give extremely low judgments.

Table S2

Quantile Regression Results from Experiment 1

Quantile	Estimate	Std.Err.	t	p
0.1	-35	4.283	-8.172	<.001
0.2	-31	9.028	-3.434	.001
0.3	-4	5.933	-0.674	.501
0.4	-2	1.418	-1.41	.160
0.5	-1	1.02	-0.98	.328
0.6	-1	0.842	-1.187	.236
0.7	0	1.219	0.00	1.000
0.8	-1	1.749	-0.572	.568
0.9	-2	2.865	-0.698	0.486

Note. The single-probability condition is coded with 0 and the multiple-probability condition with 1.

Supplement C: Percentages of Low Judgments

We preregistered for each experiment that we would inspect the distributions within the two conditions via histograms, and in case of a bimodal or heavily skewed distribution, we would repeat the test while excluding participants in the distribution on the lower scale end. As this approach is not very objective, we switched to the quantile regressions, which do not require any exclusion of participants. Based on the suggestions of an anonymous reviewer, we present the preregistered analysis below – first, the results on our experiments, and then those on Karmarkar and Kupor (2023).

Table S3

Our experiments with the preregistered exclusions

Exp. Nr.	Scenario	Outcome Probability	Low-Path Probabilities	Dependent Variable	Without Exclusions	Distribution	Excluded participants	With Exclusions	Total Outcome Probability Stated?
1	Flea	58%	Seven Probabilities (7-10%)	Slider (0-100)	$t(190) = 4.43$, $p < .001$, $d = 0.64$, 95% CI [0.35, 0.93], $BF_{10} > 1000$	Bimodality, Threshold 40/100	Single: 3/93 Multiple: 25/97	$t(162) = 0.25$, $p = .805$, $d = 0.04$, 95% CI [-0.27, 0.35], $BF_{10} = 0.17$	Yes
2a	Flea	58%	Seven Probabilities (7-10%)	Slider (0-100)	$t(390) = 6.97$, $p < .001$, $d = 0.70$, 95% CI [0.50, 0.91], $BF_{10} > 1000$	Bimodality, Threshold 40/100	Single: 6/198 Multiple: 50/195	$t(334) = 1.45$, $p = .149$, $d = 0.16$, 95% CI [-0.06, 0.37], $BF_{10} = 0.33$	Yes
2b	Flea	58%	Seven Probabilities (7-10%)	Slider (0-100)	$t(382) = 2.27$, $p = .024$, $d = 0.23$, 95% CI [0.03, 0.43], $BF_{10} = 1.34$	Bimodality, but nof after applying memory check	--	--	Yes
2c	Flea	58%	Seven Probabilities (7-10%)	Slider (0-100)	$t(381) = 0.01$, $p = .992$, $d = 0.00$, 95% CI [-0.20, 0.20], $BF_{10} = 0.11$	--	--	--	Yes
3	Vitamin	96%	21 Probabilities (2-8%)	Slider (0-100)	$F(1, 398) = 125.90$, $p < .001$	Flat	--	-- (No exclusions done)	No
4	Allergy	86%	40% + 46%	Slider (0-100)	$t(398) = 3.55$, $p < .001$, $d = 0.35$, 95% CI [0.16, 0.55], $BF_{10} = 44.33$	Slightly skewed	--	-- (No exclusions done)	On the previous page

Table S4

Re-Analysis of Karmarkar and Kupor (2023) with additional exclusions

Exp. Nr.	Scenario	Outcome Probability	Low-Path Probabilities	Dependent Variable	Original Result	Distribution	Excluded participants	Re-analysis	Total Outcome Probability Stated?
1	Flea	58%	Seven Probabilities (7-10%)	Slider (0-100)	$t(259) = 4.11$, $p < .001$, $d = 0.51$, 95% CI [0.26, 0.76], $BF_{10} = 336.84$	Bimodality, Threshold 35/100	Single: 6/136 Multiple: 30/125	$t(223) = -0.41$, $p = .682$, $d = -0.06$, 95% CI [-0.32, 0.21], $BF_{10} = 0.16$	Yes
2	Cancer	70%	Six Probabilities (67%, 1%, 4 x 0.5%)	Slider (0-100)	$t(452) = 3.62$, $p < .001$, $d = 0.34$, 95% CI [0.15, 0.52], $BF_{10} = 55.32$	Weak Bimodality, Threshold 31/100	Single: 2/224 Multiple: 9/230	$t(441) = 2.91$, $p = .004$, $d = 0.28$, 95% CI [0.09, 0.46], $BF_{10} = 6.15$	On the previous page
3A	Medication	70%/70.001%	70% + 0.001%	Slider (0-100)	$t(306) = 3.28$, $p = .001$, $d = 0.37$, 95% CI [0.15, 0.60], $BF_{10} = 20.21$	Weak Bimodality, Threshold 45/100	Single: 3/152 Multiple: 14/156	$t(289) = 1.65$, $p = .101$, $d = 0.19$, 95% CI [-0.04, 0.42], $BF_{10} = 0.47$	Yes
3B	Allergy	86%	40% + 46%	Slider (0-100)	$t(398) = 3.55$, $p < .001$, $d = 0.35$, 95% CI [0.16, 0.55], $BF_{10} = 44.33$	Slightly skewed	--	-- (No exclusions done)	On the previous page
4	Medication	58%	Nine Probabilities (3-10%)	Slider (0-100)	$t(390) = 2.89$, $p = .004$, $d = 0.29$, 95% CI [0.09, 0.49], $BF_{10} = 6.00$	Strong Bimodality, Threshold 35/100	Single: 5/193 Multiple: 38/199	$t(347) = -2.19$, $p = .029$, $d = -0.23$, 95% CI [-0.45, -0.02], $BF_{10} = 1.17$	Yes

Exp. Nr.	Scenario	Outcome Probability	Low-Path Probabilities	Dependent Variable	Original Result	Distribution	Excluded participants	Re-analysis	Total Outcome Probability Stated?
4	Medication	58%	Nine Probabilities (3-10%)	Proportion of Unlikely Thoughts	$t(383) = -2.11$, $p = .035$, $d = -0.22$, 95% CI [-0.42, -0.01], $BF_{10} = 0.96$	Extreme Floor effect of 0%. Few 100%.	Single: 6/188 Multiple: 15/197 [Excluding 100%]	$t(362) = -1.01$, $p = .315$, $d = -0.11$, 95% CI [-0.31, 0.10], $BF_{10} = 0.19$<br><br>$t(340) = 0.41$, $p = .679$, $d = 0.04$, 95% CI [-0.17, 0.26], $BF_{10} = 0.13$ (when using the other exclusion criterion)	Yes
5	Vitamin	96%	21 Probabilities (2-8%)	Slider (0-100)	$t(998) = 14.24$, $p < .001$, $d = 0.90$, 95% CI [0.77, 1.03], $BF_{10} > 1000$	Flat	--	-- (No exclusions done)	No
6	Flea	74%	Nine Probabilities (3-15%)	Choice 1- 11	$t(511) = 2.26$, $p = .024$, $d = 0.20$, 95% CI [0.03, 0.37], $BF_{10} = 1.17$	Ceiling Effect: ~ 50% pick the highest option	--	-- (No exclusions done)	No
SA	Balls in Urn	60%	Eleven Probabilities (3-8%)	Slider (0-100)	$t(301) = 4.96$, $p < .001$, $d = 0.57$, 95% CI [0.34, 0.80], $BF_{10} > 1000$	Bimodality, Threshold 35/100	Single: 8/150 Multiple: 46/153	$t(247) = -1.45$, $p = .147$, $d = -0.19$, 95% CI [-0.44, 0.07], $BF_{10} = 0.38$	No
SB1	Lottery Game	64%	Seven Probabilities (7-13%)	Slider (0-100)	$t(203) = 3.25$, $p = .001$, $d = 0.45$, 95% CI [0.18, 0.73], $BF_{10} = 19.48$	Bimodality, Threshold 35/100	Single: 3/104 Multiple: 17/101	$t(183) = 0.49$, $p = .625$, $d = 0.07$, 95% CI [-0.22, 0.36], $BF_{10} = 0.18$	Yes

Exp. Nr.	Scenario	Outcome Probability	Low-Path Probabilities	Dependent Variable	Original Result	Distribution	Excluded participants	Re-analysis	Total Outcome Probability Stated?
SB2	Lottery Game	64%	Seven Probabilities (7-13%)	Slider (0-100)	$t(301) = 2.15$, $p = .033$, $d = 0.25$, 95% CI [0.02, 0.47], $BF_{10} = 1.13$	Bimodality, Threshold 30/100	Single: 1/151 Multiple: 13/152	$t(287) = -0.34$, $p = .731$, $d = -0.04$, 95% CI [-0.27, 0.19], $BF_{10} = 0.14$	Yes
SC	Medication	60%/61%	60% + 1%	Slider (0-100)	$t(420) = 3.32$, $p < .001$, $d = 0.32$, 95% CI [0.13, 0.51], $BF_{10} = 20.89$	Bimodality, Threshold 25/100	Single: 3/210 Multiple: 18/212	$t(399) = 1.11$, $p = .266$, $d = 0.11$, 95% CI [-0.08, 0.31], $BF_{10} = 0.20$	Yes
SD	Allergy	40%	Five Probabilities (5-11%)	Slider (0-100)	$t(258) = 2.47$, $p = .014$, $d = 0.31$, 95% CI [0.06, 0.55], $BF_{10} = 2.39$	--	--	-- (No exclusions done)	No
SE	Genes	27-28%	25%, 2-3%	Slider (0-100)	$t(392) = 2.37$, $p = .018$, $d = 0.24$, 95% CI [0.04, 0.44], $BF_{10} = 1.64$	--	--	-- (No exclusions done)	No

We also present a visualization of the thought generation data (Experiment 4) and the purchase behavior (Experiment 6) below.

Figure S2

Distribution of the Proportion of Unlikely Thoughts in Experiment 4

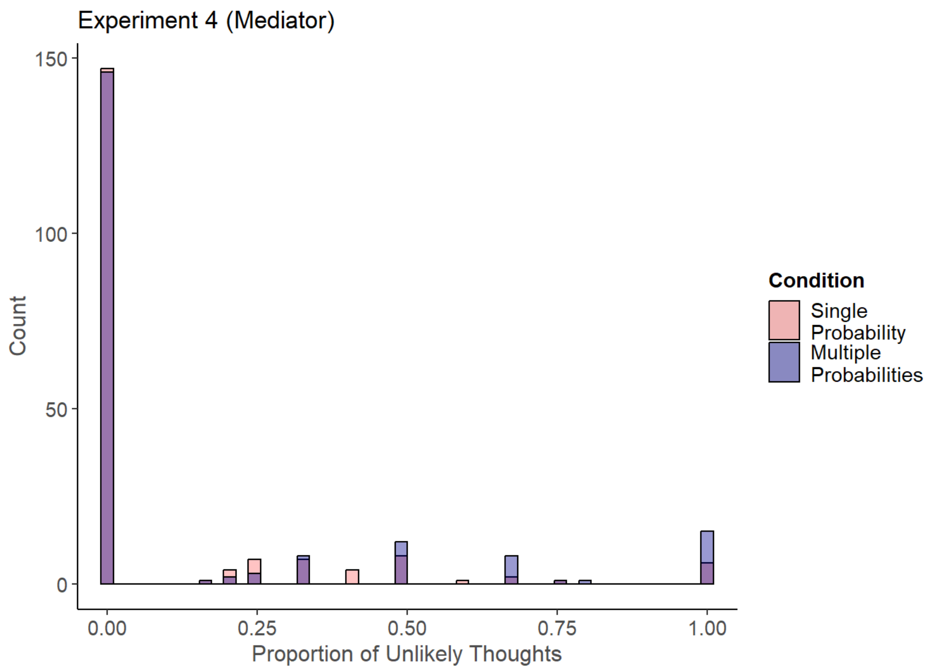

Figure S3

Distribution of the Proportion of Choices in Experiment 6

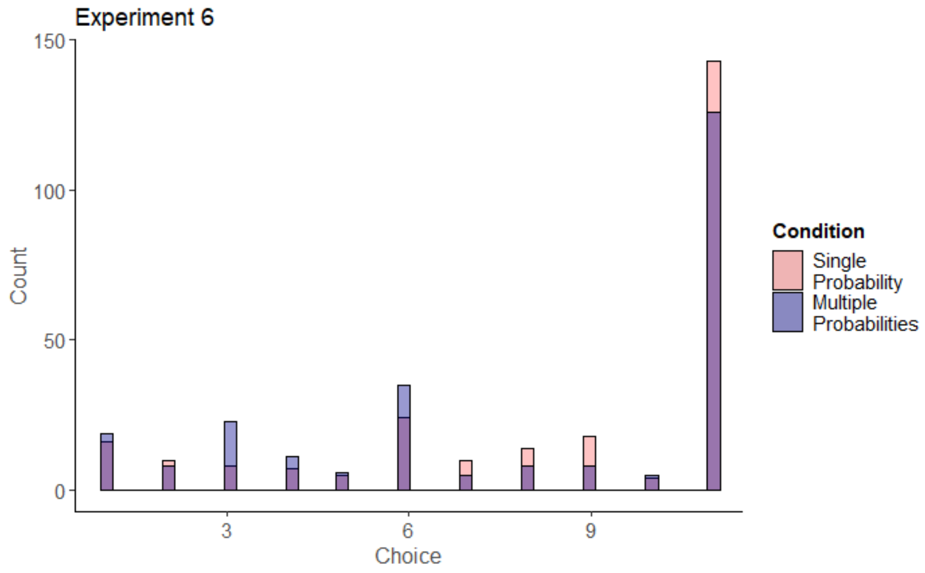

Supplement D: Additional Figures and Tables in our Experiments

Table S5

Exemplary Explanations by Participants Regarding Why and How They Made a Specific Judgment Split By Condition and Percentile Relative to Other Judgments

Percentile (by Condition)	Single Probability Condition	Multiple Probabilities Condition
.01-.10	• 58% of all people get a flea bite but only 58% of that 58% will get the infection<br>• If 58% of all people get bitten by the fly and then 58% of those people get the infection Then that's lower than 50% of people. So I went for lower than 50%	• average percentage chance of fleas causing bacterial infection<br>• the percentages accross the varients were very low. So on average from all the different fleas the possible infection is also low
.45-.55	• The text said that 58% of people were likely to get a bite that lead to the bacterial infection, so I slid the slider to approximately the same number.<br>• Figure you gave was 58% of people so I tried to select 58 on the scale	• I aimed roughly for the 58% mark as that is what was stated as the amount of people coming down with a bacterial infection after being bitten<br>• people have a 58% of being biten by a flea
.90-1.00	• I just thought that was a high enough amount of people to get bitten<br>• Because you said every single person could get a bacterial infection	• Because I consider 58% chance of developing a bacterial infection very high.<br>• If the total the various percentages, these total to a very high risk of getting bitten.

Note. All answers are provided on the OSF.

Table S6

Results from ANOVAs and Pairwise Comparisons in Experiment 1

ANOVA		*t*-test / Pairwise comparison			
		Subgroup	M_{Single} (N)	$M_{Multiple}$ (N)	Result
		Overall	59.00 (93)	48.36 (99)	$t(190) = 4.43$, $p < .001$, $d = 0.64$, 95% CI [0.35, 0.93], $BF_{10} > 1000$
Split: Awareness	Condition: $F(1, 188) = 32.93$, $p < .001$, $\eta^2_p = .149$<br>Awareness: $F(1, 188) = 13.84$, $p < .001$, $\eta^2_p = .069$<br>Condition x Awareness: $F(1, 188) = 29.31$, $p < .001$, $\eta^2_p = .135$	No Awareness	62.6 (15)	34.3 (39)	$t(188) = 6.41$, $p < .001$, $d = 1.17$, 95% CI [0.53, 1.81], $BF_{10} = 81.12$
		Awareness	58.3 (78)	57.5 (60)	$t(188) = 0.33$, $p = .741$, $d = 0.10$, 95% CI [-0.24, 0.44], $BF_{10} = 0.21$
Split: Understanding	Condition: $F(1, 188) = 26.49$, $p < .001$, $\eta^2_p = .123$<br>Understanding: $F(1, 188) = 27.08$, $p < .001$, $\eta^2_p = .126$<br>Condition x Understanding: $F(1, 188) = 13.24$, $p < .001$, $\eta^2_p = .066$	Incorrect	53.8 (13)	22.8 (34)	$t(188) = 4.62$, $p < .001$, $d = 1.61$, 95% CI [0.56, 2.64], $BF_{10} = 14.26$
		Correct	59.4 (80)	54.0 (65)	$t(188) = 2.42$, $p = .016$, $d = 0.40$, 95% CI [0.09, 0.70], $BF_{10} = 3.39$

Note. For the Understanding ANOVA, we used classifications where at least one coder indicated incorrect understanding. In line with the preregistration, we also conducted an ANOVA when coding only those responses as incorrect where both raters agreed (see the R Markdown on the OSF for detailed results). This analysis showed the same effects except that the Unlikelihood Effect was not significant anymore for participants with correct understanding.

Figure S4

Pie Chart in Experiment 2b for the Multiple-Probabilities Condition

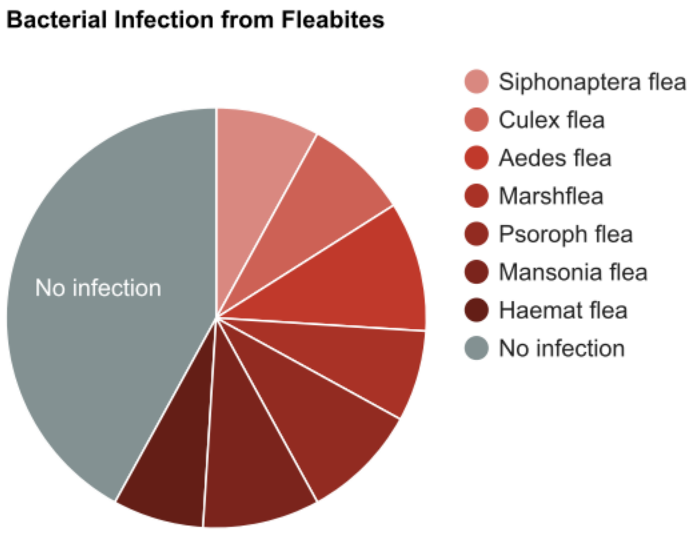

Table S7

Preregistered Tests in Experiments 2a-c

Experiment	Exclusion	M_{Single} *(N)*	$M_{Multiple}$ *(N)*	*t*	*p*	*d*	*BF10*	*Z (p)*
2a (Baseline)	--	59.5 (198)	48.8 (194)	6.97	< .001	0.70 [0.50, 0.91]	> 1000	--
2b (Pie Chart)	--	60.7 (197)	58.1 (187)	2.27	.024	0.23 [0.03, 0.43]	1.34	3.23 (.001)
	Memory Check	60.1 (171)	59.3 (129)	0.66	.508	0.08 [-0.15, 0.31]	0.16	4.01 (< .001)
2c (Math Problem)	--	58.3 (197)	58.3 (186)	0.01	.992	0.00 [-0.20, 0.20]	0.11	4.82 (< .001)
	Memory Check	57.9 (184)	58.2 (151)	- 0.28	.782	-0.03 [-0.25, 0.18]	0.13	4.85 (< .001)
	Math Problem	58.0 (177)	58.3 (145)	- 0.32	.750	-0.04 [-0.26, 0.18]	0.13	4.84 (< .001)

Note. As the experiments were collected separately, we compared the effect sizes in two ways: First, the 95% CIs from Experiments 2b/2c did not overlap with those of Experiment 2a. Second, meta-analytical *z*-tests for comparing two independent effect sizes (Borenstein et al., 2009, p. 155) showed that all effect sizes were significantly smaller than in the baseline Experiment 2a. *Z (p)* refer to *z*- and *p*-values from these meta-analytical *z*-tests. We also repeated the analyses using the combined effect size from Experiment 1 and 2a as a reference baseline. Note that the two experiments were identical up to the point when participants made their likelihood judgments; thus, a combination is justified and provides a more robust estimate for the baseline effect. The results were nearly identical and are thus only provided in the R Markdown file in our OSF folder. Note that none of these comparisons between the experiments were preregistered.

Figure S5

Likelihood Judgments and Quantile Regressions in Experiment 3

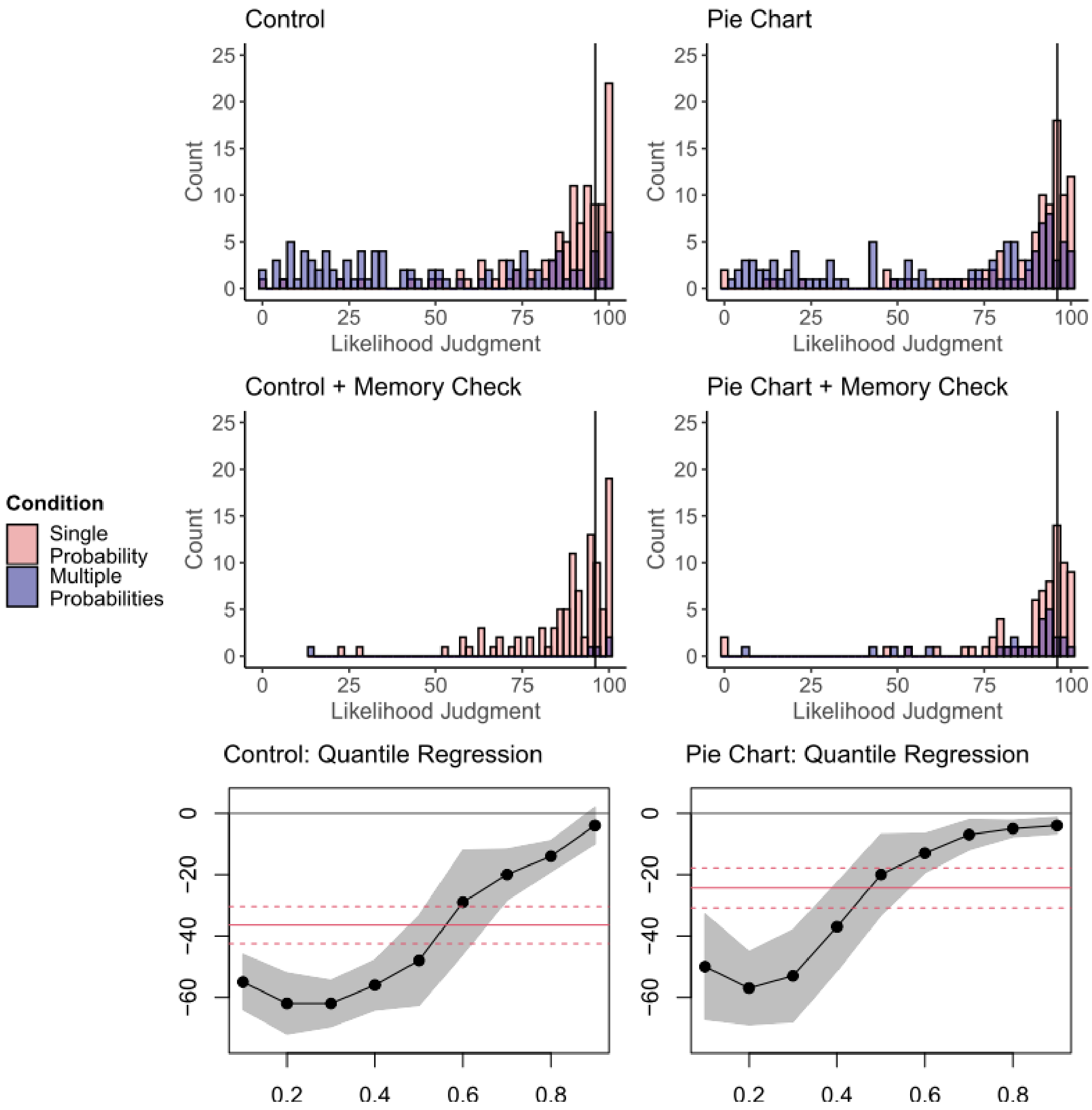

Note. The vertical line represents the total outcome probability on the subjective likelihood slider.

Table S8

Results from ANOVAs and Pairwise Comparisons in Experiment 3

ANOVA		*t*-test / Pairwise comparison			
		Subgroup	M_{Single} *(N)*	$M_{Multiple}$ *(N)*	Result
		Overall	85.2	55.0	$t(400) = 11.07$, $p < .001$, $d = 1.10$, 95% CI [0.89, 1.31], $BF_{10} > 1000$
Full Sample	Condition: $F(1, 398) = 125.90$, $p < .001$, $\eta^2_p = .240$<br>Format: $F(1, 398) = 3.52$, $p = .061$, $\eta^2_p = .009$<br>Condition x Format: $F(1, 398) = 5.01$, $p = .026$, $\eta^2_p = .012$	Control	85.7 (108)	49.2 (95)	$t(398) = 9.56$, $p < .001$, $d = 1.39$, 95% CI [1.08, 1.70], $BF_{10} > 1000$
		Pie Chart	84.7 (96)	60.3 (103)	$t(398) = 6.32$, $p < .001$, $d = 0.87$, 95% CI [0.57, 1.16], $BF_{10} > 1000$
Exclusion: 96+/-4	Condition: $F(1, 276) = 79.80$, $p < .001$, $\eta^2_p = .224$<br>Format: $F(1, 276) = 2.79$, $p = .096$, $\eta^2_p = .010$<br>Condition x Format: $F(1, 276) = 2.82$, $p = .094$, $\eta^2_p = .010$	Control	88.0 (101)	55.6 (40)	$t(276) = 7.17$, $p < .001$, $d = 1.50$, 95% CI [1.09, 1.90], $BF_{10} > 1000$
		Pie Chart	87.9 (72)	65.9 (67)	$t(276) = 6.32$, $p < .001$, $d = 0.83$, 95% CI [0.49, 1.18], $BF_{10} > 1000$
Exclusion: 96+/-4 without 100	Condition: $F(1, 200) = 2.24$, $p = .136$, $\eta^2_p = .011$<br>Format: $F(1, 200) = 0.00$, $p = .965$, $\eta^2_p < .001$<br>Condition x Format: $F(1, 200) = 0.00$, $p = .960$, $\eta^2_p < .001$	Control	88.0 (101)	81.0 (5)	$t(200) = 0.87$, $p = .388$, $d = 0.44$, 95% CI [-0.46, 1.34], $BF_{10} = 0.55$
		Pie Chart	87.9 (72)	81.4 (26)	$t(200) = 1.62$, $p = .107$, $d = 0.34$, 95% CI [-0.11, 0.79], $BF_{10} = 0.61$

Figure S6

Likelihood Judgments in Experiment 4

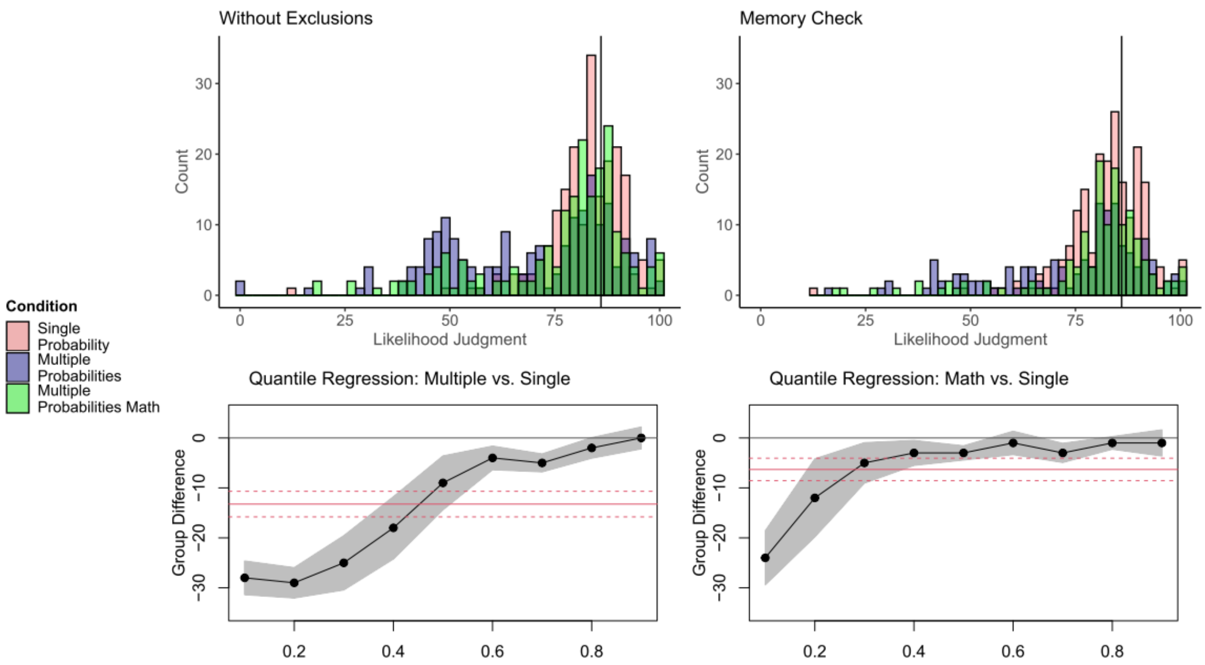

Note. The vertical line represents the total outcome probability on the subjective likelihood slider.

Table S9

Results from ANOVAs and Pairwise Comparisons in Experiment 4

	ANOVA	*t*-test / Pairwise comparison			
		M_{Single} *(N)*	$M_{Multiple}$ *(N)*	M_{Math} *(N)*	Result
Full Sample	$F(2, 598) = 34.37, p < .001, \eta^2_p = .103$	82.4 (205)	69.1 (199)	76.1 (197)	Single vs. Multiple: $t(598) = 8.29, p < .001, d = 0.85$, 95% CI [0.64, 1.05], $BF_{10} > 1000$ Single vs. Math: $t(598) = 3.94, p < .001, d = 0.37$, 95% CI [0.18, 0.57], $BF_{10} = 82.36$ Math vs. Multiple: $t(598) = 4.30, p < .001, d = 0.46$, 95% CI [0.26, 0.66], $BF_{10} > 1000$
Exclusion	$F(2, 476) = 13.18, p < .001, \eta^2_p = .052$	82.3 (203)	74.3 (138)	79.1 (165) (138 incl. Math)	Single vs. Multiple: $t(476) = 5.13, p < .001, d = 0.60$, 95% CI [0.37, 0.82], $BF_{10} > 1000$ Single vs. Math: $t(476) = 2.08, p = .039, d = 0.26$, 95% CI [0.04, 0.48], $BF_{10} = 1.35$ Math vs. Multiple: $t(476) = 2.80, p = .005, d = 0.29$, 95% CI [0.05, 0.52], $BF_{10} = 1.91$

Supplement E: Further Experiments

Experiments SA&SB

Methods

2 x 400 English native speakers from the UK and the US were recruited via Prolific Academic. As preregistered (and as done in the original experiment), we excluded all participants who failed to answer which animal the scenario dealt with, leading to the final sample sizes in Table S10. Both experiments were preregistered: https://aspredicted.org/44X_NMV, https://aspredicted.org/NG2_F72

Experiment SA – Question and Memory Check. Experiment SA was identical to Experiment 2a except for two changes: First, we replaced the question for the likelihood judgment with "In total, how likely are people to get the *newly-discovered* bacterial infection?" We suspected that adding the two words may direct participants' focus on the total outcome probability because this was also the wording in the first sentence of the task where the total outcome probability was mentioned. Second, we added had the memory check from experiment 2b.

Experiment SB – Natural Frequencies and Memory Check. Experiment 3c was identical to Experiment 3a except for two changes: First, we displayed the information in a natural-frequency format. Specifically, the information denoted was: "There is a population of 1000 people. From this population, 580 get a flea bite that causes a newly discovered bacterial infection. Specifically: 80 of the 1000 people get this bacterial infection from getting bitten by a siphonaptera flea. [...]". Second, we added the memory check for the total outcome probability (here, as a frequency) from Experiment 2b.

Results

In Experiment SA, the effect was as strong as in the baseline Experiment 2a, despite using a slightly different question wording. However, 42 participants (Single: 7, Multiple: 35)

did not report the correct total outcome probability in the memory check. Excluding these participants reduced the effect, but the effect was still significant.

In Experiment SB, where the natural frequency format was used, the effect was weaker than in the baseline Experiment 3a. Ninety-nine participants (Single: 33, Multiple: 66) did not report the correct total outcome probability (as frequency) in the memory check. Unexpectedly, excluding these participants did not reduce the effect.

Table S10

Preregistered Tests in Experiments SA/SB and 2a

Experiment	Exclusion	Mean (*SD*) Single	Multiple	*N*	*t*	*p*	*d*	*BF10*	*Z (p)*
2a (Baseline)	--	59.46 (9.68)	48.80 (19.19)	392	6.97	<.001	0.70 [0.50, 0.91]	>1000	--
SA (Question)	--	58.99 (9.16)	48.67 (19.27)	379	6.73	<.001	0.69 [0.48, 0.90]	>1000	0.08 (.936)
	Memory Check	58.80 (9.23)	54.41 (14.12)	337	3.45	<.001	0.38 [0.16, 0.59]	33.39	2.14 (.032)
SB (Natural Frequencies)	--	64.26 (13.39)	58.83 (20.22)	384	3.11	.002	0.32 [0.12, 0.52]	11.33	2.65 (.008)
	Memory Check	64.75 (13.84)	59.31 (15.85)	288	3.10	.002	0.37 [0.13, 0.60]	12.19	2.11 (.035)

Note. Z (p) refer to z and p values from meta-analytical *z*-tests for comparing an effect size to the baseline condition.

Experiment SC – Asking the Right Question

Different types of question should trigger different algebraic computations. Asking for a general likelihood judgment (“How likely are people to get the bacterial infection?”) seems to make some people compute the average of the low pathway probabilities, and others compute the sum. In Experiment SC, we asked participants two separate questions – one that specifically requires the computation of the average, and the other requires the computation of the sum. Based on our theorizing, people should show a stronger unlikelihood effect on the first than on the second judgment.

Methods

Experiment SC was identical to Experiment SA except for the dependent variable. Here, we asked participants for two separate judgments – one requiring the average and one requiring the sum of the pathway probabilities. Specifically, participants were asked "in total, how likely are people to get the infection *from a specific flea*?" and "in total, how likely are people to get the infection *from any type of flea*?" The judgments were assessed both on the same page as the probability information was shown, each with its own slider. As in the previous studies, we added the memory check for the total outcome probability. We collected data from $N = 400$ English native speakers who were UK or US citizens from Prolific Academic (266 female, 133 male, 1 prefer not to say, $M_{age} = 41.83$). Eleven participants had to be excluded because they could not name the type of animal the scenario was about. The experiment was preregistered on aspredicted.com (https://aspredicted.org/FWR_5DF).

Results

In line with the preregistration, we analyzed the judgments with a 2 (Condition: Single Probability vs. Multiple Probabilities) x 2 (Judgment: Specific vs. Any Type) ANOVA. In addition to a significant main effect of Condition, $F(1, 387) = 117.99$, $p < .001$, $\eta^2_p = .234$, there was a significant main effect of Judgment Type, $F(1, 387) = 59.00$, $p < .001$, $\eta^2_p = .132$, and a significant interaction, $F(1, 387) = 130.64$, $p < .001$, $\eta^2_p = .252$. For the specific-type judgment, there was a strong effect of condition in line with the Unlikelihood Effect, $M_{Multiple} = 26.8$, $M_{Single} = 57.4$, $t(387) = 17.41$, $p < .001$, $d = 1.77$, 95% CI [1.53, 2.00], $BF_{10} > 1000$. For the any-type judgment, there was no difference, $M_{Multiple} = 52.5$, $M_{Single} = 52.4$, $t(387) = -0.02$, $p = .985$, $d = -0.00$, 95% CI [-0.20, 0.20], $BF_{10} = 0.11$.

Five participants in the single-probability and 50 participants in the multiple-probabilities condition did not report the correct total outcome probability. Following the preregistration, we repeated the analysis without these participants. This did not change the significance of any effect, or systematically alter the distributions (see Figure 5).

Figure S7

Likelihood Judgments in Experiment SC

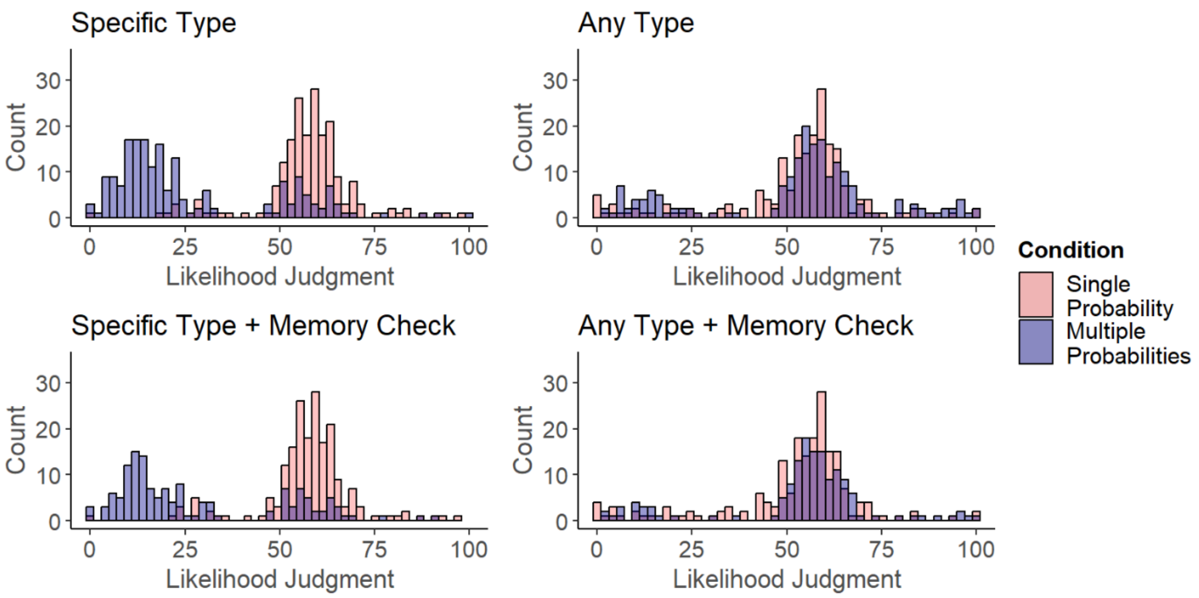

Discussion

Experiment SC further supports our idea that the Unlikelihood Effect is partially driven by people's lack of processing the relevant information. Asking a question that required averaging the pathway probabilities yielded a strong Unlikelihood Effect. However, no effect was found in a question format that required the computation of the sum.
